# Language teachers’ wellbeing in Scotland: challenges and coping pathways

**DOI:** 10.3389/fpsyg.2026.1816586

**Published:** 2026-06-04

**Authors:** Manuel Jesús Cardoso-Pulido, Francisco Valdera-Gil, Raúl Ruiz-Cecilia, Hazel Crichton

**Affiliations:** 1 Department of Language, Literature and Education, School of Educational Sciences, University of Granada, Granada, Spain; 2 School of Education, University of Glasgow, Glasgow, United Kingdom

**Keywords:** coping strategies, ecological systems theory, language learning and teaching and assessment washback, Scotland, target language use, teacher agency, teacher wellbeing

## Abstract

This study examines language teachers’ wellbeing in Scotland through a holistic ecological framework, addressing how wellbeing is shaped by challenges and coping pathways across interacting systems of practice and policy. Drawing on Bronfenbrenner’s ecological systems theory, the research investigates the interplay between microsystem (classroom relations and pedagogy), mesosystem (collegial and external support), exosystem (school leadership, workload, curriculum and policy enactment), macrosystem (societal ideologies and national frameworks), and chronosystem (career stage and post-COVID change) layers. Data were generated through in-depth semi-structured interviews with nine primary and secondary language teachers. Approximately 80,000 words of interview data were analysed using a hybrid inductive–deductive thematic analysis, with psychological and social capital conceptualised as cross-cutting resources influencing wellbeing across systems. Findings show that teachers’ wellbeing was strongest when they could enact an “ideal teacher-self,” characterised by positive classroom rapports, meaningful communicative pedagogy, creative use of culture and projects, and confident target language use. Conversely, wellbeing was eroded by chronic workload and administrative demands, misalignment between curriculum, assessment, and pedagogy, particularly with high-stakes examinations and the marginal status of languages within schools and a predominantly monolingual societal ideology. Supportive departmental cultures, peer mentoring, collaborative school environments, fair and communicative leadership, and external social support emerged as key protective factors, while isolation and top-down decision-making intensified vulnerability. Across the chronosystem, more experienced teachers described a gradual development of boundary-setting and self-care strategies, with early-career teachers and those facing job insecurity reporting heightened vulnerability. Post-COVID schooling brought increased digital competence but also persistent pressures related to learners’ foundational skill gaps and expanded bureaucratic demands, underscoring the need for multi-level approaches to sustaining language teachers’ wellbeing.

## 1 Introduction

Teacher wellbeing has become a growing concern in educational research, particularly in light of increasing workload intensification, accountability pressures, emotional demands, and teacher attrition. In language education, these concerns are compounded by domain-specific factors such as target language use, communicative pedagogy, intercultural work, and the variable status of languages within school systems. As a result, language teacher wellbeing cannot be adequately understood as a purely individual matter, but must instead be examined in relation to the professional, institutional, and sociocultural environments in which teachers work.

In this study, teacher wellbeing is understood as a dynamic and context-sensitive state shaped by the interaction of personal, relational, professional, and structural conditions that support or constrain teachers’ sense of purpose, agency, satisfaction, and capacity to teach well. This understanding is consistent with research that conceptualises wellbeing as multifaceted, contextually mediated, and subject to change over time rather than as a fixed or exclusively individual trait (Dodge et al., 2012; Mercer, 2021; McCallum et al., 2017). While debates persist around hedonic and eudaimonic views of wellbeing (Deci and Ryan, 2008; Zeng and Chen, 2020), the present study adopts an ecological understanding that foregrounds the interplay between subjective experience and contextual conditions.

This ecological perspective is especially relevant in language education. Previous research has shown that language teachers’ wellbeing is shaped not only by general professional demands such as workload, leadership, collegiality, and accountability, but also by more specific pressures related to language teaching itself, including language anxiety, communicative competence expectations, intercultural demands, and the status of the subject being taught (Horwitz, 1996; Borg, 2006; Mercer, 2021; Mercer and Murillo-Miranda, 2025). Recent scholarship has further argued that teacher wellbeing is best understood through multi-layered socio-ecological frameworks that account for the interaction of individual, relational, institutional, and policy-level influences (Jin et al., 2021; Durrani and Makhmetova, 2025; Sadeghi and Pourbahram, 2024). Rather than locating wellbeing solely within the teacher, these approaches show how it emerges through teachers’ ongoing negotiation of affordances and constraints across their working environments.

The present study draws on Bronfenbrenner’s ecological systems theory (Bronfenbrenner, 1979) as its principal conceptual framework. This model offers a useful basis for examining how wellbeing is shaped across interconnected layers of experience. In this study, the microsystem refers to teachers’ immediate professional settings, such as classroom interaction, pedagogy, and relationships with learners and colleagues. The mesosystem concerns the connections across settings, including collaboration within and beyond departments, communication across school structures, and support networks extending outside school. The exosystem includes institutional and organisational conditions that shape teachers’ work indirectly or structurally, such as leadership, timetabling, workload, curriculum enactment, and local policy mediation. The macrosystem refers to broader sociocultural and political forces, including ideologies about languages, national educational priorities, and assessment regimes. Finally, the chronosystem captures temporal dimensions, such as career stage, professional transitions, and post-pandemic change.

Building on this ecological framework, the notions of psychological and social capital are used as sensitising concepts to help interpret some of the resources teachers draw on when responding to ecological pressures. Psychological capital refers to individual resources such as resilience, hope, optimism, and self-efficacy (Youssef-Morgan and Luthans, 2015), while social capital concerns trust, support, reciprocity, and recognition within professional and personal networks (Portela et al., 2013). These constructs do not function here as alternative theoretical frameworks, but as supporting concepts that illuminate how teachers navigate ecological conditions. The study also acknowledges plurilingual wellbeing as a relevant domain-specific dimension of language education, especially where teachers’ and learners’ linguistic repertoires shape classroom experience (Sugranyes Ernest et al., 2024).

The Scottish context offers a particularly relevant setting for such an investigation. Teachers working in the Scottish context face a number of challenges. Although spending per child is greater than in England (Sibieta and Snape, 2025), recent cuts to the number of teachers and importantly, support staff for children with additional needs [Educational Institute of Scotland (EIS) and EIS-FELA, 2014] means that teachers are having to respond to ‘excessive workload demands’ [Educational Institute of Scotland (EIS), 2015]. A report commissioned by the Educational Institute of Scotland, a teachers’ union, found that teachers regularly worked over 11 h per week, unpaid, to stay on top of their workload (Hulme et al., 2024), revealing tensions between teachers’ working conditions and their feelings of self-efficacy (Ainsworth and Oldfield, 2019).

In Scotland, the teaching profession is overseen by the General Teaching Council for Scotland (GTCS), an independent self-regulatory body. Teacher education is conceptualised as a two-phased scheme. Upon completion of a teaching qualification, the Scottish Government guarantees newly qualified teachers (NQTs) a one-year post in a state school to complete a Teacher Induction Scheme (TIS). GTCS data show that between 4.11 and 5.69% of registered teachers left the profession each year between 2016 and 2020. In 2022/23, 16% of NQTs left during or after induction, while in 2023/24 the figure rose to 18%. Examining teacher wellbeing is therefore important not only in relation to teachers’ lived experiences, but also in relation to attrition and the support that can be provided during initial and early-career teacher education.

Research on wellbeing in language education remains comparatively limited, although several studies have examined language teaching in the Scottish primary sector (Crichton and Templeton, 2010; Valdera Gil and Crichton, 2020; Crichton, 2021, 2018; Kanaki, 2025). Existing work has highlighted challenges such as language anxiety, perceived linguistic inadequacy, intercultural demands, and the status of languages within schools (Horwitz, 1996; Sercu, 2006; Borg, 2006; Guijarro-Ojeda et al., 2021; Mercer and Murillo-Miranda, 2025). Kubanyiova (2012) and Valdera Gil (2020) applied the teacher-selves theory to study the processes of teacher development in communicative language teaching with language teachers in Slovakia and Scotland. Both studies took an ecological approach to teacher agency. Findings linked teacher development with wellbeing. Teachers described moments, often taking place in the same day, in which they became the ‘ideal-teacher self’ they had always wanted to enact, or performing duties they felt they had to do, ‘ought-to-teacher self’. At times, with a more detrimental effect to their wellbeing, they became their ‘feared teacher-self’ when they had to carry out duties which in their views took them away from what really mattered for learning.

At the same time, the Scottish educational sphere is shaped by Curriculum for Excellence, which provides a curriculum framework for learners aged 3 to 18 (Education Scotland, n.d.), and by the 1 + 2 policy, through which the Scottish Government recommends that all learners study two languages in addition to their mother tongue (Scottish Government, 2012a, 2012b). Although this policy was welcomed when first introduced, previous research has identified persistent difficulties in its implementation, including uneven provision, lack of dedicated time, limited teacher confidence, and the competing prioritisation of other initiatives (Valdera Gil and Crichton, 2018; Kanaki, 2020).

Although teacher wellbeing has received increasing attention in recent years, and ecological approaches are becoming more prominent in language education research, there remains limited qualitative work examining how language teachers’ wellbeing in Scotland is shaped across interacting ecological systems. In particular, there is a need for research that traces not only the challenges teachers face, but also the coping pathways and protective factors they mobilise across classroom, school, policy, and societal levels. This matters because teacher wellbeing is not only consequential for teachers themselves, but also for the sustainability of language education and for the capacity of teachers to enact meaningful, communicative, and inclusive pedagogies.

This study therefore examines the wellbeing of primary and secondary language teachers in Scotland through an ecological lens. Drawing on in-depth semi-structured interviews with nine teachers, it addresses the following research questions:

RQ1: What are the links and the interplay between ecological subsystems and the experiences of language(s) teachers within the realities of Scottish schooling?
RQ2: What are the key challenges and coping mechanisms reported by language(s) teachers in Scotland in relation to their wellbeing?

## 2 Methodology

The data presented is developed as a part of a larger funded research project, by the Spanish Ministry of Science and Innovation (code: PID2021-1283410B-100), that sought to study language teachers’ wellbeing across different contexts.

### 2.1 Participants

Nine teachers based in Scottish secondary and primary schools volunteered to take part in semi-structured interviews. Their teaching experiences spanned from less than a year to more than 30 years. Although the range of experience may seem at first sight rather large, the authors were keen to gain as much information from as wide a range of sources as possible, in order to present as full a picture as possible about teachers’ perceptions of wellbeing at each stage in their career. Participants were recruited through direct contact by members of the research team based in Glasgow. A purposive sampling approach was used to identify teachers who were actively teaching languages in Scottish primary or secondary schools and whose taught language(s) were not their first language. This criterion was considered relevant because the study sought to explore wellbeing in relation to language teaching, including target language use, perceived linguistic competence, and professional identity.^1^ The sample was gender-balanced, with four male and five female teachers. Detailed data can be observed in Table 1.

**Table 1:** Participants’ information.

Participant	Years teaching	Teaching position	Languages taught
P1.	24–30	Secondary	Spanish, French, Italian
P2.	0–3	Primary	Spanish, French
P3.	16–23	Secondary	French, German
P4.	8–15	Secondary	French, Spanish
P5.	4–7	Primary	French, Spanish
P6.	+ 30	Secondary	French, German, Spanish
P7.	4–7	Secondary	French, Spanish
P8.	4–7	Secondary	French, Spanish, Italian
P9.	0–3	Secondary	Spanish, French

### 2.2 Data collection

To gather data in the most comprehensive manner, an in-depth interview was designed with open questions, so that participants could express the depth and nuances of the matters at hand. The review of existing literature guided the process of determining the common questions and variables in this field of knowledge, especially the works of Ryff (1989) and Ryff and Keyes (1995). The interview schedule was reviewed by members of the research team before data collection to ensure that the questions were clear, non-leading, and aligned with the study aims. Minor adjustments were made to wording and sequencing before the interviews were conducted.

All participants were asked the same core prompts, although curriculum-related questions were adapted depending on whether participants worked in primary or secondary education, given the different curricular arrangements in each level. The semi-structured format also allowed the interviewers to use follow-up questions to clarify participants’ responses, invite them to expand on specific experiences, and explore issues that emerged naturally during conversation (Rubin and Rubin, 2005). As can be deduced from the full interview included in the Appendix, the main themes covered were:

- Aspects related to language(s) teachers’ practical and evaluative dimensions, such as beliefs (cultural theme), relationships and power (structural theme), and resources (material theme)
- Aspects related to language(s) teachers’ interactional dimensions, such as epistemologies and teachers’ identities
- Aspects related to language(s) teachers’ projective dimensions, such as their current and future pedagogical repertoire

The interviews were conducted by members of the research team with experience in language teacher education and qualitative interviewing. The interviewers were not members of the participants’ schools and did not hold managerial or evaluative roles in relation to them. This was intended to reduce potential power imbalances and encourage participants to speak openly about their experiences (Rubin and Rubin, 2005). All interviews were conducted in person in the participants’ own schools and classrooms after the school day, with a view to ensuring a familiar and comfortable environment. The interviews were conducted in English, which was participants’ first language, and lasted approximately 75 min per participant. Interviews began with background questions about participants’ teaching experience and professional trajectory, before moving to questions about language teaching practices, target language use, school relationships, institutional resources, and perceived wellbeing. The final part of the interview invited participants to reflect on future professional aspirations and the kinds of support they considered necessary. With participants’ consent, interviews were audio-recorded and subsequently transcribed verbatim. Transcripts were not returned to participants for member checking; however, they were reviewed internally by the research team for accuracy, and all identifiable information was anonymised prior to analysis. The full interview schedule reflected the broader funded project; however, the present article draws specifically on the interview material related to teachers’ professional experience, wellbeing, ecological conditions of work, and coping.

With respect to ethical considerations, prior to collecting data, ethics approval was obtained from the relevant partner universities. As well as adhering to the guidelines recommended by the partner universities, care was taken to adhere to the British Educational Research Association’s guidelines for non-clinical research involving human participants (BERA, 2024). Participants were given a participant information sheet, a privacy note, and a consent form to sign and were informed that they could withdraw from the study at any point. To ensure data confidentiality, a number was assigned to each participant (P1, P2, etc.) and any identifiable data were anonymised. All recordings were stored on password-protected computers and destroyed upon the completion of the transcription.

### 2.3 Data analysis

The study employed thematic analysis (Braun and Clarke, 2006) on a corpus of over 80,000 words of interview transcripts, which were read multiple times to gain an overall understanding. Coding was conducted in NVivo (Version 12), beginning with an initial descriptive round (Saldaña, 2016) to identify data relevant to the research questions. Inductive coding was complemented by a deductive approach based on themes from the literature (Proudfoot, 2022). Both recurring patterns and distinctive individual emotional experiences were considered. A second round of coding refined the initial codes, and data extracts were reviewed to ensure each theme represented a coherent pattern. Each member of the research team coded the data individually before discussing the themes in a group forum, in order to ensure that the findings could be considered trustworthy (Cornish et al., 2014).

Agreement among coders was not assumed at the outset. After individual coding, the research team met to compare coding decisions, discuss discrepancies, and refine the thematic structure. Differences mainly concerned the placement of particular extracts within ecological subsystems and the boundaries between them. These differences were mitigated through collective discussion, return to the original transcripts, and revision of code definitions until agreement was reached. This process was necessary to enhance the coherence and transparency of the interpretive thematic analysis.

## 3 Findings

In line with Bronfenbrenner’s ecological approach adopted in this study, the table below summarises the themes associated with each subsystem, together with the values that increased or eroded participants’ wellbeing. The themes show that teachers’ wellbeing is relational and contextual, not individual. The values increasing or eroding wellbeing are underpinned by psychological capital (hope, optimism, self-efficacy) and social capital (trust, support, reciprocity, recognition) (Table 2).

**Table 2:** Summary of the main findings.

Sub-system	Themes	Values increasing wellbeing	Values eroding wellbeing
3.1 Microsystem	3.1.1 Classroom rapport & influence on students	Feeling they are a good example; close rapport with students (P1, P2, P3, P5, P6, P7)	Emotional exhaustion with pupil disengagement (P1, P2, P5, P9)
	3.1.2 Classroom milieu, communicative competence and high stakes examinations	Sense of mastery when routines work and sense of autonomy (P3, P7, P9)	Large class-sizes and misbehaviour (P1, P2, P3, P5, P7, P9)
	3.1.3 Alignment in curriculum, assessment and pedagogy	Freedom in designing tasks involving culture and projects (P2, P3, P4, P5, P6, P8)	Exam formats and unrealistic topics (P1, P2, P3, P5, P7, P9)
	3.1.4 Target language use	Using the TL confidently and extensively (P1, P3, P4, P6, P7, P9)	Language anxiety and self-questioning when teaching another additional language (P2, P5, P8, P9)
	3.1.5 Life goals, teacher-selves (identity as language teachers)	Seeing oneself as “the teacher I want to be” and long-term commitment to languages (All except P4)	Fear of being “the teacher I do not want to be” due to the perceived low status of the profession (P2, P4, P7, P8, P9)
3.2 Mesosystem	3.2.1 School cohesion & peer-mentoring	Feeling listened to and constructive networks within the school staff (P1, P3, P6, P7, P9)	Downgrading of languages priorities and resources (P2, P5, P8, P9)
	3.2.2 Department cohesion & peer-mentoring	Feeling part of a team with shared values, space for mentoring and asking for help (All except P4)	Feeling of isolation (P4)
	3.2.3 Outside school support	Feeling of support from family and friends, emotional balance (P1, P3, P4, P5, P6, P8, P9)	Limited or absent external support (P2, P7)
3.3 Exosystem	3.3.1 Scottish curriculum & language policy	Sense of being linked with European objectives and plurilingual aims (P1, P4, P6)	High-pressure from exams and the lack of coherence between theory and practice (P2, P3, P5, P7, P9)
	3.3.2 Status of languages	Promotion, celebration and visibility of languages (P3, P7, P9)	Priority of other subjects such as literacy, numeracy and STEM (P1, P2, P5, P6, P8)
	3.3.3 School leadership and agency	Fair and distributive with clear communication and a sense of being trusted (P1, P3, P6, P7)	Top-down decisions with lack of clarity and high expectations (P2, P5, P8, P9)
	3.3.4 Workload & administrative paperwork	Ability to be organised (P3, P6)	Work overload and extensive paperwork leading to emotional exhaustion (All)
3.4 Macrosystem	3.4.1 Societal support for languages & ideology	Recognition of the cultural value of languages (P1, P4)	Students questioning its usefulness because they already speak English (P2, P3, P5, P6, P7, P8, P9)
	3.4.2 National policy and framework	Focused on communicative and European approaches (P4, P6)	Memory-based assessment; disjunction between assessment and real language use (P1, P3, P5, P7, P8, P9)
	3.4.3 Expectations about teachers	Feeling valued by some families and communities, especially after COVID (P3)	High expectations, feeling they “have to do everything” (All except P3)
3.5 Chronosystem	3.5.1 Professional path and agency	Growth from past experiences to be able to “switch off” and stronger sense of agency (All except P2)	Anxiety and vulnerability due to overwork, feeling of “failing” (P2, P5, P8, P9)
	3.5.2 Post-COVID adaptation	Sense of pride after coping with pandemic issues and increased digital skills (P3, P6, P8)	Gaps in students’ literacy skills and increased paperwork, sense of isolation from government initiatives (P1, P2, P4, P5, P7, P9)

### 3.1 Microsystem

The microsystem comprises teachers’ immediate teaching environment, including their classrooms, direct interactions with pupils, and daily instructional practices. Across participants, this level emerged as both the most rewarding and the most demanding sphere of their professional lives. Five interrelated themes were classified: (a) classroom relationships and the influence of students; (b) the classroom milieu; (c) teaching praxis and curriculum fit; (d) target language use; and (e) life goals and identity as language teachers.

#### 3.1.1 Classroom rapport and influence of students

All participants consistently considered pupil-teacher rapports as the most immediate source of fulfilment in their work. P1 spoke of “the relationships with pupils, being able to inspire them, every day there’s something to laugh about,” while others referred to “light bulb moments” when learners finally understood how language(s) works, describing these as the best part of the job (P2, P3, P4). P8 established this as a wellbeing trigger and especially rewarding when students’ confidence is boosted when being able to hold a basic conversation in the target language. P9 indicated that languages provide “something tangible they take away from school.” Likewise, P2 emphasized the symbiotic relationship between students and teachers and how satisfaction works in a language classroom “seeing the children get excitement out of actually ‘languaging’ or ‘doing the language’. Nonetheless, teachers underlined the emotional cost of pupil disengagement. Many noted that “you spend so much energy trying to persuade them that languages matter, and when they shut down, it’s exhausting” (P9), while P4 and P7 described how a small group of reluctant learners can impact the atmosphere in the class.

#### 3.1.2 Classroom milieu, communicative competence and high stakes examinations

The classroom milieu was portrayed as intense and, at times, overwhelming. Participants described language lessons as highly demanding for teachers, requiring continuous target language provision, interaction, and feedback, alongside constant movement and sustained cognitive effort. P5 explained that “you are on your feet the whole time, and by the end of the day your head is still buzzing.” Large class sizes, especially in secondary schools, compounded this sense of pressure, making it difficult to organise practical and communicative work with more than 30 learners (P1, P3), especially if the content of the exam is not compatible from communicative perspectives. “they can talk about colours but cannot order a *croissant*.” (P4).

Post-COVID behavioural changes added further complexity, as some pupils struggled more with concentration and routines (P8, P9). However, when behaviour was settled and routines held, teachers experienced the classroom as a stimulating space: “When the class is calm, everything flows; we can actually teach” (P3).

#### 3.1.3 Alignment of curriculum, assessment and pedagogy

Participants showed strong commitment to creative, meaningful teaching, often integrating cultural content and interdisciplinary tasks/projects. P4 highlighted the satisfaction of “bringing a subject alive… seeing the penny drop […] especially when pupils could talk about real things, not just colours”. Similarly, P5 described integrating Spanish into numeracy so that pupils would engage with it and enjoy it, which, in turn, made her own teaching more enjoyable. Yet, again, this creative drive frequently collided with curricular and assessment constraints, showcasing a perceived gap between topics and real-life use.

In the secondary classroom, P3 and P7 linked this misalignment to the pressure which exams bring to communicative pedagogy and the curriculum learners engage with in the classroom. There was a perception that the exam rewarded memorisation rather than genuine communication.

#### 3.1.4 Target language use

Target language (TL) use emerged as a marker of professional identity but also as a site of vulnerability. For some teachers, speaking the TL extensively in class was described as when they felt most like themselves as a teacher (P1, P4, P6, P7, P9). P3 associated her enjoyment with seeing pupils actually using the language rather than working predominantly in English. P1 noted that although pupils were initially shocked when she used the TL, “they come to enjoy it.” Others, particularly when teaching an additional language or new level, reported their own perceived reduced linguistic competence. In this sense, P8 felt “only half a step ahead” when teaching another language and tended to second-guess himself, while P2 and P5 recalled lacking confidence in spontaneous TL use in an additional language earlier in their careers.

#### 3.1.5 Life goals and teacher-selves

With regards to the teacher ideal-self, many participants framed their work as language teachers in terms of long-standing life projects and personal identity, underpinned by the teacher they had conceptualised they always wanted to be. P5 remembered that she had “always said [she] wanted to be a teacher to change the world,” which she now connects to opening linguistic and cultural horizons for her students. Similarly, P6 explained that “languages shaped who I am; teaching them feels natural,” and P3 described teaching as “what I was meant to do.” For P9, language classrooms had previously been a “safe place with an open-minded [and] kind teacher,” and this experience now shapes his commitment to creating similar spaces. These narratives present language teaching as a way of enacting deeply held values of care, inclusion and social justice. Conversely, identities of teachers could be unsettled when the subject was marginalised or practice felt misaligned with beliefs. P2 voiced concern about becoming the teacher she did not want to be when exam pressures overshadow communicative approaches.

### 3.2 Mesosystem

The mesosystem captures relationships and interactions across overlapping contexts and structuring teachers’ wellbeing. Three themes were identified: (a) school cohesion and peer mentoring; (b) departmental cohesion and peer mentoring; and (c) outside-school support.

#### 3.2.1 School cohesion and peer mentoring

Participants described the wider school culture as an important layer of support and protection and, occasionally, tension. Several participants emphasised the benefits of working in schools where collaboration is encouraged across departments and levels (P1, P3, P6, P7, P9). This sense of being part of a cohesive staff group contributed to feeling listened to and recognised. Others in secondary and primary settings (P2, P5, P6, P7, P9) similarly highlighted how shared planning, regular meetings, and informal mentoring from more experienced colleagues made them feel that they were a team rather than isolated practitioners P3 declared “management encourages collaboration.” These responses highlight that trust, openness and shared leadership enhance collective identity.

At the same time, the quality of school cohesion was uneven. Some participants pointed out moments when languages “do not count the same as other subjects” (P5) or when decisions are made without taking into account the language department (P8), generating a sense of marginalisation and limiting their perceived influence on school priorities.

#### 3.2.2 Department cohesion and peer mentoring

Within languages departments in particular, collegiality was repeatedly described as a core protective factor for wellbeing. Many teachers depicted their departments as safe spaces for professional and emotional exchange. P2 commented that in her context “we do lots together, planning… we are a team […] there’s no silly questions, you can go to anybody to talk about anything.” Likewise, P9 stated that in his school almost everybody feels like family, while P7 described his department as a united front, underlining shared passion for languages and collective responsibility for pupils.

In addition, P9 stressed collaborative practices, like treasure hunts, food tastings, and CPD for staff, as positive activities for wellbeing. Some participants took part in activities which ranged from weekly planning meetings and shared Google Drive folders (P1, P3, P5, P8) to reciprocal support around technology or new initiatives. P5, for instance, described helping an older colleague with digital tools and joked that it made her feel listened to and nurtured, capturing humour and mutual learning. In contrast, P4 recalled earlier experiences as a single-person department and feeling completely isolated, which she had found hard and creatively draining.

#### 3.2.3 Outside-school support

Support beyond the school also played a significant role in preserving teachers’ wellbeing. Several teachers referred to partners, family, and friends as essential sources of emotional balance (P1, P3, P4, P5, P6, P8, P9). P5 explained that her family helps her switch off and reset, while P8 noted that friends outside education keep things in perspective, providing an alternative frame of reference, non-school related. P1 described her husband and children as really good emotional support, indicating that these relationships helped her manage the demands of teaching alongside family life. Not all participants experienced this support to the same extent, as P7 hinted at periods in which limited external networks made her feel as though “work takes over.”

### 3.3 Exosystem

The exosystem refers to institutional and organisational conditions considering teachers’ work beyond the classroom, including school leadership, workload structures, the status of languages, and the ways in which Scottish curriculum and language policies are enacted in practice.

#### 3.3.1 Scottish curriculum and language policy

Teachers’ accounts of Curriculum for Excellence (CfE), the 1 + 2 policy, and national examinations revealed a complex matrix in which policy ideals and classroom realities only partially align. Some participants (P3, P4, P6) valued the flexibility of CfE and the aspirations of 1 + 2, describing the policy as “fantastic in theory” (P4) due to its alignment with broader European frameworks, such as plurilingualism and pluriliteracies or intercultural development.

However, most participants marked significant gaps in implementation and support. P2 noted that although 1 + 2 appears in the school improvement plan, it had never been discussed at CPD sessions. P5 affirmed that “policies arrive without real support – you are left to figure it out yourself.” High-stakes exam structures were perceived as particularly misaligned with communicative aims. P7 commented that “the exams push us backwards; they are not reflective of real communication,” and P5 similarly observed that “we talk about communication, but exams still reward memorisation.” These comments link back to the perceived lack of alignment of curriculum, assessment and pedagogy. These inconsistencies generated frustration and disillusionment and were experienced as a key constraint on teacher agency and wellbeing, especially since P7 affirmed “without passionate language teachers working beside you, it is difficult to get pupils engaged.” In this sense, P9 declared “it would be nice to be recognised for all the extra things we do, teachers feel undervalued”.

#### 3.3.2 Status of languages

The organisational status of modern languages within Scottish schools emerged as a recurring concern. Several participants reported that languages were often the first subject to lose timetable space or senior leadership attention (P1, P2, P5, P7, P8, P9). In this sense, P7 observed that “the first thing to go in budget cuts are language classes,” and P8 commented that languages are pressed between perceived bigger priorities. P6 highlighted that the Brexit situation hindered the status of modern language subjects and colleagues. Such patterns contributed to feelings that ML is structurally undervalued and sidelined, which in turn affected motivation and professional agency.

#### 3.3.3 School leadership

Participants described leadership practices as a crucial trigger of wellbeing. In several cases, distributed models of responsibility were experienced as empowering (P1, P3, P4, P5, P6, P9). For example, P6 noted that “everyone’s encouraged to have some leadership responsibility… it’s not too hierarchical,” while P3 and P4 highlighted opportunities for staff to make decisions collectively in their departments. Such approaches fostered trust and reinforced teachers’ sense of agency.

However, others pointed to limitations in consultation and communication. P8 described how messages from senior leaders could be “hard to understand exactly what’s expected,” and P2 linked the absence of a clear language leader: “we have someone for literacy, but not for languages, even though languages is literacy as well” to feelings that the subject lacked visibility and validity.

#### 3.3.4 Workload and administrative paperwork

Workload and administrative demands were universally identified as major stressors. Teachers across contexts referred to never having enough hours in the day (P1, P2, P3, P5, P7, P9) and to the cumulative effect of reports, parents’ evenings, assessments, and transition work: “there’s always something to stress about” (P5); “most of the stress does not come from teaching but from everything else” (P1); “March is Mad March, workload is not balanced” (P7). Although some participants mentioned occasional periods where planning felt manageable (P3, P6), the dominant pattern was one of systemic chronic overwork, with paperwork and accountability requirements drawing time and energy away from pedagogical and relational aspects of the job.

### 3.4 Macrosystem

The macrosystem refers to broader cultural, political and policy environments that form how languages and teachers are valued in Scotland, participants’ accounts converging around three themes: societal support for languages and prevailing ideologies, national policy and assessment frameworks, and wider expectations about teachers.

#### 3.4.1 Societal support for languages and ideology

Across interviews, teachers situated their work within a predominantly monolingual culture. Several participants (P2, P4, P7, P8, P9) referred to a recurring message that students and parents echoed “we speak English, so what’s the point of learning another language?” (P2). Others contrasted the Scottish context with countries where learning additional languages is often taken for granted: “In Spain everyone wants to learn English… here pupils ask why they need Spanish” (P7). These comments suggest that broader societal ideologies about English as sufficient undermine the perceived relevance of language learning and, indirectly, teachers’ sense of professional agency.

#### 3.4.2 National policy and framework

Participants expressed ambivalent views about the Scottish language policy. Many appreciated the intentions behind Curriculum for Excellence and the 1 + 2 approach, describing the policy as a good idea (P4, P5, P7, P8) emphasising its potential to promote plurilingualism. However, teachers also pointed to gaps between these aspirations and their practical application. P5 noted that “it’s a good idea, but not implemented effectively… I see schools with only one language instead of two.” Others drew attention to the optional nature of languages in the Senior Phase (15 to 18) and the dominance of high-stakes exams which in their opinion were very memory-based: “National 5 is entirely memory-based… are pupils really learning a language?” (P7). These concerns echo the issue of lack of alignment of curriculum, assessment and pedagogy, which seems to underpin different ecological layers.

#### 3.4.3 Expectations about teachers

P1 noted that “parents and pupils expect teachers to be on call all the time” with digital communication extending availability beyond the school day. Others described an evolution in public discourse during and after the COVID-19 pandemic. P3 recalled that during lockdown teachers were praised for their hard work, but felt that this recognition was short-lived, with teachers once again expected to parent and solve a range of social issues. Several participants remarked that people do not realise how skilled language teachers are and still see the subject as boring or secondary to STEM (P2, P5, P9). These perceptions contribute to a sense that teachers’ work is both highly demanding and under-recognised, silently undermining their experience of wellbeing within a wider social landscape.

### 3.5 Chronosystem

The chronosystem captures the temporal dimension of teachers’ wellbeing, that is, how their professional lives shift over time and other major events such as COVID-19 and post-COVID adaptation.

#### 3.5.1 Professional path and agency

All participants showed a clear temporal pattern from early-career overload to more deliberate boundary-setting, balance and a stronger sense of agency. Several teachers described their probationary induction period as particularly intense (P2, P3, P5, P8, P9). P5 recalled that her probation year was chaotic, and P2 admitted that, as a student-teacher, she used to work weekends, whereas now she has learned to stop at six and not work on weekends. Over time, many reported learning to switch off and protect their non-working time, whether by going to the gym (P1, P8), walking the dog (P4), and/or muting notifications outside school (P9). For some, career moves were also part of long-term research for meaningful impact, P6 described leaving one post and then moving into teacher education to make a bigger difference, while P4 retrained from science into languages once she realised languages were her calling.

#### 3.5.2 Post-COVID adaptation

Teachers also reflected on the enduring imprint of the COVID-19 period as a temporal backdrop to their current wellbeing. Nonetheless, the pandemic period resulted in some participants gaining greater digital confidence and new repertoires (P1, P4, P8), “COVID forced us to get good with digital tools” (P8). However, the main emphasis in the interviews was placed on gaps in students’ basic knowledge of literacy. P2 observed that students are not meeting the expected standards, while P7 and P4 echoed this, stating pupils who missed crucial primary years, have a deficit in foundational knowledge and now encounter additional challenges in language acquisition. P9 further emphasized the need to rebuild the foundation from scratch. These accounts indicate that, despite teachers’ adaptations and professional growth, they are operating within a prolonged period of readjustment. The pandemic’s ongoing consequences continue to influence classroom demands and, consequently, their perception of wellbeing.

## 4 Discussion

### 4.1 What are the links and the interplay between ecological subsystems and the experiences of language teachers within the realities of Scottish schooling?

The findings indicate that language teachers’ wellbeing in Scotland is best understood as an ecological phenomenon shaped across interacting systems rather than as an individual condition alone. Participants’ accounts showed that wellbeing emerged through the interplay of classroom relationships and pedagogy (microsystem), collegial and external support (mesosystem), institutional conditions such as leadership, workload, and policy enactment (exosystem), wider societal ideologies and assessment priorities (macrosystem), and career-stage and post-pandemic developments over time (chronosystem). This supports previous ecological research showing that teacher wellbeing is produced through dynamic interactions across professional, relational, and structural levels rather than residing solely within the individual teacher (Price and McCallum, 2015; McCallum et al., 2017; Mercer, 2021; Jin et al., 2021).

At the microsystem level, participants’ wellbeing was strongest when they could establish positive relationships with learners, engage in communicative pedagogy, use the target language confidently, and feel that their teaching aligned with their professional values. These findings are consistent with previous research showing that classroom climate, relational trust, and pedagogical authenticity are central to teacher wellbeing in language education (MacIntyre et al., 2019; Mercer, 2021; Mercer and Murillo-Miranda, 2025). However, these same classroom spaces also became sites of strain when pupil disengagement, large class sizes, behavioural pressures, and language anxiety undermined teachers’ sense of efficacy and fulfilment.

A particularly important finding concerns the lack of alignment between curriculum, assessment, and pedagogy. Participants repeatedly described tension between communicative approaches to language teaching and the demands of examination systems that reward memorisation and narrow performance. This suggests that classroom experience cannot be understood in isolation from broader structures. Pressures felt in the microsystem were often the local expression of exosystem and macrosystem conditions, especially policy enactment, assessment regimes, and accountability expectations. In this sense, the study extends previous work on washback and communicative language teaching by showing that the consequences are not only pedagogical, but also affect teachers’ wellbeing and agency (Barnes, 2016; Cheng and Curtis, 2004; Hayward, 2023, 2015; Xi, 2025).

The mesosystem emerged as an important protective layer. Departmental cohesion, peer mentoring, collaboration across the school, and support from family and friends all contributed to teachers’ emotional balance and professional resilience. These findings echo earlier studies showing that relational trust, collegiality, and supportive school cultures are central to teacher wellbeing (Aelterman et al., 2007; Johnson et al., 2014; Yin et al., 2016). In the present study, such support was especially significant where teachers felt that languages were structurally marginalised, suggesting that relational resources can partly buffer wider institutional pressures.

At the exosystem level, school leadership, workload, and the enactment of Curriculum for Excellence and the 1 + 2 policy played a major role in shaping teachers’ wellbeing. Fair, communicative, and distributive leadership was experienced as empowering, while unclear expectations and top-down decision-making reduced teachers’ sense of agency. At the same time, workload and administrative demands were described almost universally as major sources of emotional exhaustion. These findings are consistent with research indicating that wellbeing is affected not only by classroom practice but also by the organisation of teachers’ work, the mediation of policy, and the degree of institutional support available (Forde et al., 2021; Kim and Asbury, 2020; Mairi, 2023).

The macrosystem was visible in participants’ accounts of the broader status of languages in Scotland. Teachers frequently described a predominantly monolingual social ideology in which English was perceived as sufficient and additional languages were often treated as less essential than literacy, numeracy, or STEM. These ideological conditions shaped how teachers perceived the status and legitimacy of their subject, and in turn influenced their motivation and sense of professional worth. This supports previous arguments that language teacher wellbeing is shaped not only by immediate school conditions but also by wider cultural and political discourses about the value of languages (Sercu, 2006; Crichton, 2021; Mercer and Murillo-Miranda, 2025).

The chronosystem adds an important temporal dimension to these findings. Participants’ accounts suggested that wellbeing changes over time, with more experienced teachers often reporting stronger boundary-setting strategies, greater perspective, and a more developed sense of agency, while earlier-career teachers described greater vulnerability, overload, and uncertainty. In addition, post-COVID schooling remained an important temporal backdrop, with participants reporting both increased digital confidence and continuing concerns about learners’ literacy, concentration, and foundational knowledge. These patterns suggest that teacher wellbeing is not static, but evolves through career stage, professional change, and wider historical events.

These findings can also be read through the teachers’ possible selves (Kubanyiova, 2012; Valdera Gil, 2020). Most participants referred to the teacher they aspired to be in relation to values, the importance of learning languages and cultures and rapports with students. This contrasted with the teacher they felt compelled to become under conditions of examination pressure (alignment of curriculum, assessment and pedagogy), workload intensification, policy enactment or reduced subject status.

### 4.2 What are the key challenges and coping mechanisms reported by language(s) teachers in Scotland in relation to their wellbeing?

The findings show that the main challenges reported by participants were not solely personal, but ecological in nature. Chronic workload, administrative intensification, language anxiety, policy-practice misalignment, weak recognition of languages, and uneven institutional support were all described as recurrent pressures on wellbeing. At the same time, participants identified several coping pathways, including positive relationships with learners and colleagues, supportive leadership, family and friendship networks, self-care routines, and the gradual development of professional boundaries over time. The extent to which these factors protected wellbeing depended largely on the degree of agency teachers were able to exercise across the different ecological layers of their work.

Some of the challenges arising from the teachers’ experiences in this study were also perceived as opportunities to enhance their wellbeing if teachers were able to enact their agency to that end. In this sense, positive rapports with learners and colleagues, the professional comfort of the engagement of learners in language learning, or feeling valued by school leaders and/or wider society, had an effect on the participants’ wellbeing. The extent of the positive effect on wellbeing depended upon the teachers’ agentic space to ‘be’ their ideal-teacher self in the interplay of the many ecological subsystems layers. The importance of team work in any context as a contributor to wellbeing is underlined by research (Mattessich et al., 2001; Borrego et al., 2013) and clearly contributed to the teachers’ feelings of being valued.

Self-care and work-life balance seemed to be an important coping mechanism for the teachers in the study, along with the ability to see the ‘bigger picture’ and the role they played in education. In this sense, the majority of participants expressed *doing sports* as a strategy to draw psychological capital, acting as self-regulator, as teachers move throughout their chronosystem, gradually learning to set boundaries and reinterpret their whole role.

Additionally, one of the teachers involved had not secured a permanent position at the time of the interview and this seemed to have a negative effect on her wellbeing. Beyond this participant, the years of teaching experience did not seem to have a correlational effect on the participants’ wellbeing perceptions. It is important to bear in mind that at times, in this kind of qualitative studies, the voices of the burn out teachers or the teachers who do not believe in the 1 + 2 policy, may not be ‘present’ as they may not have desired to get involved in such a study.

The teachers in this study felt accomplished when they were able to be their ideal teacher-selves (Kubanyiova, 2012; Valdera Gil, 2020) and when their plurilingual wellbeing (Sugranyes Ernest et al., 2024) felt valued or when they were able to have agentic space with their learners to enact it. This happened when curriculum, assessment and pedagogy aligned which led learners to languaging practices and to develop their intercultural communicative competence as well as using their languages for learning, not only for passing exams. In turn, when it was felt that language learning was only about exams (in the secondary) or when language learning did not take place in the primary because of a cluttered curriculum, the teachers reported the negative effects this had on their wellbeing. These experiences highlight how microsystem processes (students *doing the language* and engaging plurilingually) are strongly affected by exosystem constrains (exam-driven practices, crowded timetables) and by macrosystem priorities, reinforcing the need to consider teacher wellbeing as emerging from the dynamic interplay of all ecological layers.

### 4.3 Limitations and future research directions

This study has several limitations that should be acknowledged. First, the sample was small and context-specific, comprising nine primary and secondary language teachers working in Scotland. Second, participants volunteered to take part, which means that the study may not fully capture the perspectives of teachers who are experiencing severe burnout, who feel disengaged from language policy, or who may not have had the time or willingness to participate. Third, the study relies on self-reported interview data, which provide rich insight into teachers’ perceptions and lived experiences but do not include classroom observations, documentary analysis, or longitudinal follow-up. Finally, although the ecological framework allowed the analysis to trace interactions across multiple systems, the boundaries between subsystems were sometimes fluid and blurred, reflecting the complexity of teachers’ professional lives rather than fixed analytical categories.

Future research could build on this study by exploring language teachers’ wellbeing in Scotland through larger and more diverse samples, including teachers from different local authorities, school types, career stages, and language specialisms. Longitudinal designs would be particularly valuable to examine how wellbeing develops across the chronosystem, especially in relation to early-career transitions, post-COVID educational change, workload intensification, and evolving curriculum and assessment reforms.

Further research could also compare primary and secondary language teachers more systematically, given the different curricular expectations, levels of language specialisation, and forms of institutional support in each level. In addition, future studies could examine how psychological capital, social capital, and plurilingual wellbeing interact over time, and how targeted professional development, mentoring, and school-level language policies may strengthen teachers’ agency and capacity to sustain communicative, inclusive, and meaningful language education.

### 4.4 Conclusion

This study has shown that the wellbeing of language teachers in Scotland is best understood as an ecological phenomenon shaped by the interaction of classroom experience, collegial relationships, institutional structures, policy conditions, societal ideologies, and temporal change. Teachers’ wellbeing was strongest when teachers had agency to become their ideal teacher self and use communicative pedagogy, target language, develop supportive relationships and enact their professional values. Teacher wellbeing was weakest when workload, assessment pressures, low subject status, and policy-practice gaps constrained their agency. By examining both challenges and coping pathways through an ecological lens, the study contributes to current work on language teacher wellbeing and highlights the need for multi-level responses in teacher education, school leadership, and language policy if language teaching is to remain sustainable and meaningful.

## Data Availability

The raw data supporting the conclusions of this article will be made available by the authors, without undue reservation.

## Footnotes

1 In Scotland, primary teachers teach all subjects including languages. They can qualify as teachers by undertaking a full degree, or a degree which includes a Master’s, or by undertaking a one year Professional Graduate Diploma in Education (PGDE, from now on) specialising in primary teaching after any undergraduate degree. Secondary language teachers usually have a degree in languages before undertaking a PGDE.
