# Supplementary material for: Language teachers’ wellbeing in Scotland: challenges and coping pathways
Source: Front Psychol. 2026 Jun 4;17:1816586. doi: 10.3389/fpsyg.2026.1816586 (PMC13275247; doi:10.3389/fpsyg.2026.1816586)
Supplement: Supplementary file 1 [file Supplementary_file_1.docx]

**APPENDIX**

INTERVIEW QUESTIONS

**THEME 1. AUTONOMY**

1. How would you describe the influence other people exert on you? Why?

o In the workplace (Primary/Secondary).

o Outside the workplace.

o Evolution over time.

1. Could you describe the aspects that make you feel safe or unsafe when giving your opinion in a group? Or What makes you feel confident or insecure when giving your opinion in a group?

o In the workplace (Primary/Secondary).

o Outside the workplace.

o Evolution over time.

1. How do you think others see you?

o In the workplace (Primary/Secondary).

o Outside the workplace.

o Evolution over time.

1. How do the opinions that others hold of you influence you, and why?

o In the workplace (Primary/Secondary).

o Outside the workplace.

o Evolution over time.

**THEME 2. ENVIRONMENTAL MANAGEMENT**

1. What are your strengths as a teacher? In what sense have those strengths aided you in your profession?
2. Could you describe your ideal work-life balance?
3. In what sense does your job satisfaction influence your personal life outside the workplace?
4. Which are the main sources of satisfaction in your life as a teacher and in your tasks? Which are the main sources of erosion or dissatisfaction?
5. Do you consider the teaching profession to be stressful? Why? When? How do you cope with that stress?
6. How do you rate your level of self-discipline in your life and job? Are you too hard on yourself?

**THEME 3. SELF-ACCEPTANCE**

1. Are you satisfied with what you have achieved in your life? Why? Will you consider making any changes?

o In the workplace (Primary/Secondary).

o Outside the workplace.

o Evolution over time.

1. Are you happy with who you are and how you are? Why? How would you describe yourself in general terms? (ask for sense of humour if not manifested).

o In the workplace (Primary/Secondary).

o Outside the workplace.

o Evolution over time.

1. Have you had models in the field of your initial teacher education that have had an influence on your teaching identity? And on your identity as a teacher of modern languages? In what sense or extent? (ask for LGBTIAQ+ models if part of the rainbow family).

**THEME 4. PERSONAL GROWTH**

1. Are you open to new experiences in your life, even if they are contrary or possess a conflict to your values?

o In the workplace (Primary/Secondary).

o Outside the workplace.

o Evolution over time.

1. Do you consider that your life has been a learning, changing and growing process?

o In the workplace (Primary/Secondary).

o Outside the workplace.

o Evolution over time.

1. What role do you think the modern language(s) that you teach has played in your professional and personal growth? And what about your ML teachers?
2. How important is your work in your life? (Guide in terms of: creativity, stimulation, sense of life, interest, challenges, independence, enjoyment, etc.)

**THEME 5. POSITIVE RELATIONSHIPS**

1. Do you feel loved (emotional affection) / wanted (valued)?

o In the workplace (Primary/Secondary).

o Outside the workplace.

o Evolution over time.

1. What qualities do you consider other people highlight about you?

o In the workplace (Primary/Secondary).

o Outside the workplace.

o Evolution over time.

1. To what extent are you satisfied with your personal relationships?

**THEME 6. PURPOSE IN LIFE**

1. Do you think that speaking other languages opens new horizons for your future?

o In the workplace (Primary/Secondary).

o Outside the workplace.

o Evolution over time.

1. How do you believe you are influencing your students (academically / ethically / emotionally, etc.)? What do you think is the most important thing you can teach/offer your students?
2. Do you have defined goals in life? Which?

o In the workplace (Primary/Secondary).

o Outside the workplace.

o Evolution over time.

1. Do you consider that you have been lucky in life?
2. Do you believe that what you do has value and is worth it?
3. What is your opinion on the legislation and educational acts related to modern languages?

**THEME 7: PERCEIVED PHYSICAL AND MENTAL/EMOTIONAL HEALTH**

1. How do you assess your state of physical, mental, or emotional health at this moment?

o Delve into if health limits personal and professional matters.

1. What do you do to maintain your physical and mental/emotional health?
2. Do you consider that being a modern language teacher had an impact in your physical and mental health?
3. Do you consider yourself lucky/privileged in your role as a teacher (primary/secondary)?

**THEME 8: MATERIAL WELLBEING**

1. How do you rate your socio-economic status power as a teacher (primary/secondary)?

**THEME 9: METHODOLOGY**

1. How would you define your methodology in modern languages classes?
2. At times teachers, combine a strategy CLT and CLIL have you ever had a shot at this? What are the and weaknesses of these classes?
3. How do you develop the teaching materials?
4. How do you approach diversity in ML classrooms?

**Would you like to add anything else that hasn’t come up in the interview so far?**
